# Recent Progress in Microfluidic Models of the Blood-Brain Barrier

**DOI:** 10.3390/mi10060375

**Published:** 2019-06-05

**Authors:** Lili Jiang, Shu Li, Junsong Zheng, Yan Li, Hui Huang

**Affiliations:** 1 Department of Clinical and Military Laboratory Medicine, Army Medical University, Chongqing 400038, China; jianglili@tmmu.edu.cn (L.J.); zhengalpha@tmmu.edu.cn (J.Z.); yanli@tmmu.edu.cn (Y.L.); 2 Department of Microbiology, Army Medical University, Chongqing 400038, China; lishu72@tmmu.edu.cn

**Keywords:** blood-brain barrier, microfluidic, microenvironment, in vitro model

## Abstract

The blood-brain barrier (BBB) is a critical physical and chemical barrier that maintains brain homeostasis. Researchers in academia and industry are highly motivated to develop experimental models that can accurately mimic the physiological characteristics of the BBB. Microfluidic systems, which manipulate fluids at the micrometer scale, are ideal tools for simulating the BBB microenvironment. In this review, we summarized the progress in the design and evaluation of microfluidic in vitro BBB models, including advances in chip materials, porous membranes, the use of endothelial cells, the importance of shear stress, the detection specific markers to monitor tight junction formation and integrity, measurements of TEER and permeability. We also pointed out several shortcomings of the current microfluidic models. The purpose of this paper is to let the readers understand the characteristics of different types of model design, and select appropriate design parameters according to the research needs, so as to obtain the best experimental results. We believe that the microfluidics BBB models will play an important role in neuroscience and pharmaceutical research.

## 1. Introduction

**Figure 1:**
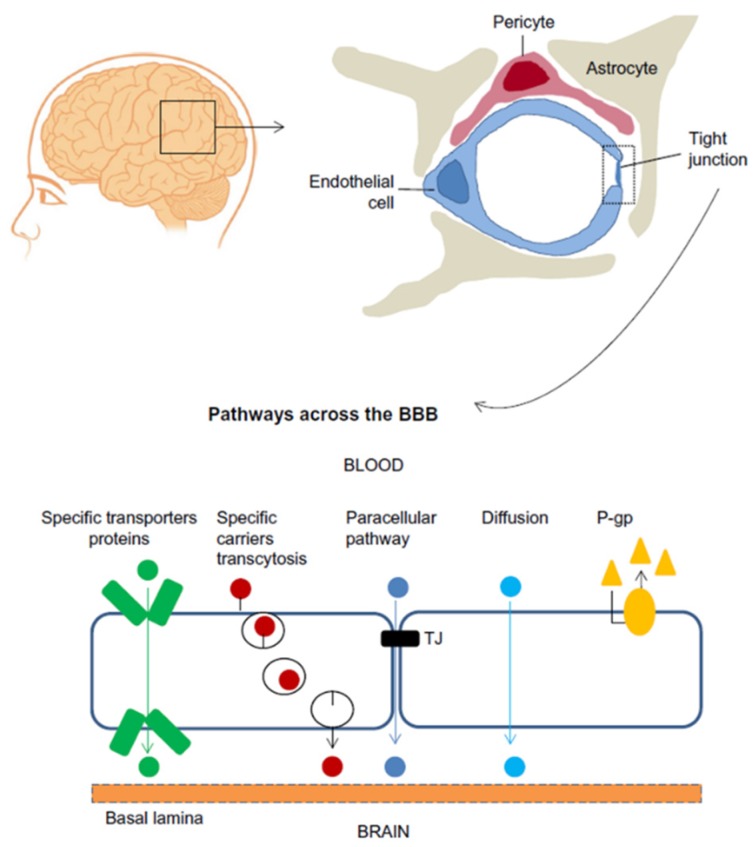
Cellular constituents of the blood-brain barrier (BBB). The BBB is formed by brain microvascular endothelial cells (BMECs), which are connected by tight junctions. The endothelium, together with the basal lamina, pericytes, and astrocytic end-feet forms the neurovascular unit. Some substances diffuse freely into and out of the brain parenchyma, others such as nutrients need specific transporters, while molecules such as insulin, leptin and transferrin are transported by receptor- mediated transcytosis. (Reprinted from Reference [11] in accordance with the Creative Commons Attribution).

The blood-brain barrier (BBB) is a highly selective barrier that regulates passive and active transport between the brain parenchyma and peripheral blood [1,2]. The BBB plays a vital role in maintaining the physical and chemical homeostasis of the central nervous system (CNS), and protects the CNS from harmful molecules and pathogens in the blood [3,4]. The BBB is a complex dynamic physiological structure network, the molecular basis that underlie BBB function depend on close interactions between adjacent brain microvascular endothelial cells (BMECs). These cells form a layer that is tightly sealed by a junctional complex composed of tight junctions and adherens junctions [5,6]. Tight junctions, which play an essential role in maintaining BBB integrity, are structures formed by at least three different types of transmembrane proteins, such as occludin, claudin and junctional adhesive molecule [7,8]. BMECs, along with neurons, pericytes, astrocytes, microglial cells, and extracellular matrix, constitute a functional network known as the neurovascular unit [9,10], Figure 1 show the cellular constituents and transport pathways of the BBB [11]. Due to the tight barrier, a number of drugs are unable to penetrate the BBB and produce therapeutic effects in the CNS [12]. Disruption or dysfunction of the BBB has been linked to a wide range of neurological disorders, including brain tumors, epilepsy, ischemic stroke, Alzheimer’s disease, and multiple sclerosis [13,14,15,16,17].

In order to gain in-depth understanding of the BBB structural and functional properties, to access the permeability of different drugs, chemicals and compounds through the BBB, studies of the BBB have become a high priority for pharmaceutical companies and biological researchers. Due to the complex structures, it is very important to establish suitable models in research [18,19]. In vivo animal models, in vitro cell-based models, and computer models are used to explore the BBB structures and functions. In vivo animal models can reflect the real state of the microenvironment, and are considered as the golden standard in research, but animal experiments are time-consuming and costly. Transwell chambers are the most widely used in vitro cell-based models, transwell models are easy to operate and reproducible, but they are static culture models and cannot reflect fluid characteristics of the BBB.

As one of the most advanced technologies, microfluidic chips use microfabrication technology to produce all kinds of micron-scale chips. In the microfluidic chips, microchannels networks are formed. By regulating the flow of liquid in microchannel, biological, and chemical analysis are performed in the chips [20,21]. In recent years, many researchers use microfluidic chip to construct the BBB models and achieved good experimental results. In this review manuscript, we discussed the technical and operational details in model design, fabrication, evaluation and application, so that we can choose appropriate models and parameters according to the research purposes in future studies.

## 2. Current Experimental Blood-Brain Barrier (BBB) Models

There is great interest in developing experimental models that mimic the well-organized and unique properties of the BBB. Ideal BBB models should have the same cell types and distribution compared with in vivo structure, can well express enzymes, receptors and transporters, can well simulate the material transport process and pathway, have high selective permeability to different substances, and have high trans-epithelial electric resistance (TEER) values [22]. Here, we will introduce various models in the BBB research.

### 2.1. In Vivo Models

Researchers first used in vivo BBB models to perform experiments in living organisms in a normal and intact state. In these models, function is studied using methods such as intravenous injection, brain perfusion, positron emission tomography, and microdialysis sampling [23]. The main advantage of in vivo models is that they provide an experimental environment that closely mimics the complexities of human physiology. The entire experiment occurs under natural conditions and can generate large amounts of reliable data [24]. However, no animal model can faithfully reproduce all of the manifestations of human disease, and thus these models must be interpreted as an approximation of human biology, limited to particular regions or other features. The most important disadvantage of in vivo models is the difficulty in translating the results they generate to the human context. More than 80% of results obtained from animal models do not correspond in a straightforward way to human responses [25]. Animal-to-animal variability is another problem. In addition, in order to observe the whole process of disease development, separate animals must be used at different stages; making experiments expensive due to labor and animal costs. Finally, during in vivo experiments, high doses of chemicals are often used. These doses are not suitable in high throughput screens for drug discovery [26].

### 2.2. In Vitro Models

In vitro models include cell culture models, brain slice models, fiber-based dynamic in vitro BBB (DIV-BBB) models, and microfluidic models.

In vitro cell culture models based on cultured cells have been used for decades to study various mechanisms that support the BBB physiology. By using the cells such as BMECs, astrocytes, and pericytes, experiments can be conducted under carefully controlled conditions. Cell culture technology is simple, with good reproducibility, and suitable for high-throughput screening. As a result, cell culture models have become important tools in the BBB research.

The most common in vitro models are the transwell models, in which one or more cell types are cultured on semi-permeable microporous inserts [27]. According to the cell types used in the experiment, it can be divided into single endothelial cell monolayer model and co-culture model. The endothelial cells monolayer model is the simplest transwell model, but it lacks the interaction with other cell types, which is crucial for the properties and functions of the BBB. In co-culture experimental models, BMECs and other cells are cultured on different sides of the membrane. Usually endothelial cells are cultured on the upper side to form luminal layer. Other cells, such as astrocytes and/or pericytes, are cultured on the lower compartment to form abluminal layer [28].

From a practical point of view, transwell models are user friendly and cost-effective. They make it possible to easily manipulate experimental conditions, such as temperature and compounds concentration. The systems can reduce experimental animal numbers, increase test speeds, cut down reagent and chemical consumption, thus facilitating moderate-throughput drug permeability screening [29]. However, transwell models are usually unable to replicate key characteristics of the BBB. First, the endothelial cells are not subjected to dynamic mechanical stimuli that can cause subtle differences in cell morphology and barrier permeability when compared with in vivo models [30]. Second, transwell models are simplified co-culture systems that do not capture the complex architecture of the BBB.

Brain slice models are also used in the BBB studies. In these models, organotypic hippocampal slices are cultured on a membrane surface and used to study the BBB functions under different physiological and pathological conditions [31,32]. All cell types and interactions are present in brain slices, so these systems provide complete structures that are excellent tools for biological and pharmacological research. However, neither fluorescent immunostaining of biomarkers, nor measurement of TEER, is convenient in slice models.

Transwell models and slice models are static models because there is no fluid flow in these systems. Fluid flow is thought to subject endothelial cells to shear forces, and these are critical for proper endothelial polarization and tight junction formation [33]. Without dynamic flows, the integrity of the barrier is affected. In static models, the TEER is much lower than in vivo models. In vivo resistance across the BBB is about 1500–8000 Ω·cm^2^, while the TEER of static in vitro models typically reaches only 150–200 Ω·cm^2^ [34]. These models also exhibit high permeability to marker molecules, and suffer from low expression and impaired functionality of P-glycoprotein efflux pump transporters, which dramatically limits their utility for drug screening.

Recently, DIV-BBB models have been developed to mimic dynamic flow. In these models, endothelial cells and other cells are cultured on the inner and outer walls of hollow fibers. Culture medium is pulsated through the tube, generating shear stress on the surface of the endothelial cells. Due to the shear stress, tight junction protein expression is increased. In these models higher TEER values and lower permeability are obtained, and the systems can better imitate the BBB environment [35]. In spite of these advantages, the DIV-BBB model has not yet been widely used in experiments due to several shortcomings. First, nine to 12 days are required to reach the maximum TEER value. Second, it is difficult to observe changes in the endothelial cells during the course of an experiment [36], and can only be inferred by consumption of glucose or production of lactic acid. Third, the thickness of the fiber wall (150 μm) is much larger than that of the porous membrane (10 μm), reducing the contact between endothelial cells and other cells [37].

In recent years, microfluidic chips have been used to construct the BBB structures, and have become increasingly popular. We will discuss the progress of these highly promising in vitro models later.

### 2.3. Computer Models

With computer aided drug design and delivery as a starting point, computer models have also been developed in the BBB research. Computer models typically build quantitative structure-activity relationships for the BBB permeation, and predict drug permeability according to the physical and chemical properties of compounds, such as van der Waals volume, topological polar surface area, lipophilicity, hydrogen-bond donator and acceptor [38,39]. Computer models are generally applied to small molecular compounds and are excellent for high throughput drug studies. However, they are applicable only to permeability prediction based on drug structure, and cannot provide information about the effectiveness of a drug or whether the drug itself affects permeability of the BBB. Nearly all results obtained using computer simulation must be verified by in vivo experiments [40].

## 3. Microfluidic In Vitro Models

Recent advances in micro-electro-mechanical systems (MEMS) technology make it possible to create microfluidic devices to mimic biological microenvironments in vitro [41,42,43]. Microfluidics is considered both a science and a technology that focuses on the behavior and manipulation of fluids at the micrometer scale [44]. Microfluidic chips require micro-scale engineering technologies to construct channels, chambers, and valves on silicon, glass, quartz, or macromolecule polymer material to form sub-micrometer sized mechanical channel structures [45]. We can perform precise and complicated operations with sub-millimeter scale fluids as desired in these microstructures using micropumps or microvalves. Various functional units can also be integrated to carry out purification, separation, and detection, making it possible to perform a series of experiments and analyses on a single chip. A microfluidic chip with these features is also called a micro-total-analytical system or a lab-on-a-chip device [46]. Microfluidic chips have potential applications in chemistry, physics, biology, medicine, and other disciplines.

The channels in microfluidic chips have similar width scales in comparison to biological systems, such as cells, macromolecules, and small organisms. Through simple or sophisticated channel design, it is possible to control the flow in microfluidic channels conveniently and accurately. Microfluidic technologies have recently been used to create three-dimensional (3D) cell co-culture devices and organ-on-a-chip systems, setting the stage for simulating the activities and responses of entire organs and organ systems under physiological or pathological conditions [47]. In 3D cell culture microfluidic chips, perfusion-based media are used to supply nutrients to the cells and remove metabolic wastes. By precisely controlling the physical and chemical microenvironment around the cells, the systems are well suited for studying biological interactions down to the cell and molecular levels, and have great potential for investigating cell-cell interactions. In recent years, with notable developments having been made in areas of bio-printing, researchers have increasingly applied bio-printing technology to the processing of microfluidic chips. As a digital manufacturing technology, bio-printing uses computer-aided design (CAD) software to accurately construct complex objects, and adds materials to 3D model data layer by layer to create microfluidic chips. Compared with conventional microfluidic chip fabrication methods, bio-printing can create more complex, uniform and reproducible architectures [48]. Microfluidic devices are evolving rapidly, and it is now possible to design microphysiologic organ-on-a chip models for guts, lungs, kidney, and other organs [49,50].

Since the microfluidic platform offers precise control of fluid transport at microscale dimensions and makes multifunctional integration possible, the microfluidic platform has been used to simulate the BBB microenvironment in various experiments. Microfluidic models clearly provide a promising platform for studying the mechanism of the BBB and evaluating CNS drugs.

### 3.1. Design of Microfluidic Models

**Figure 2:**
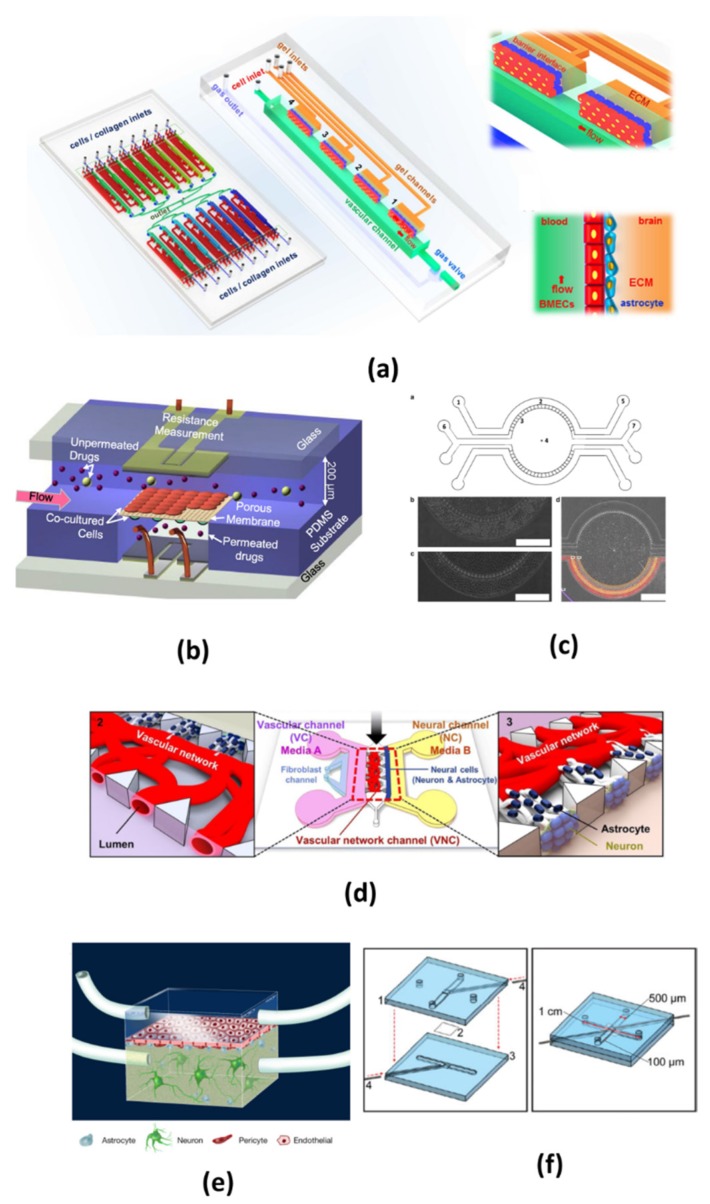
Typical design of microfluidic blood-brain barrier (BBB) in vitro models. (**a**) The chip is composed of 16 independent functional units. Each unit consists of four uniform BBB regions to mimic the BBB. The two pictures on the right show enlarged view and side view of the barrier regions consisting of brain microvascular endothelial cells (BMECs), astrocytes and 3D ECM under flow. (Reprinted from Reference [36] in accordance with the Creative Commons Attribution). (**b**) Multi-layered channel structure made from patterned PDMS substrate with dynamic flows, co-cultured cells and two sets of electrodes (Reprinted from Reference [58] with permission). (**c**) The chip is consisted of four channels, two central gel regions for co-culturing astrocytes and neurons, two side channel for hosting endothelial cells and medium. There are 3 μm pores to allow diffusion of media and tracer between the central and outer compartments, astrocytes and HUVECs were cultured in the central and outer compartment (Reprinted from Reference [59] in accordance with the Creative Commons Attribution). (**d**) In this chip, neuron and astrocytes are co-cultured in a vascular network, a system of two separate media microchannels is employed to independently emulate highly localized internal and external vascular microenvironments (Reprinted from Reference [60] in accordance with the Creative Commons Attribution). (**e**) This is a typical two-chamber system divided by a porous polycarbonate membrane. (Reprinted from Reference [61] in accordance with the Creative Commons Attribution). (**f**) A polycarbonate membrane is used to separate the top channel from the bottom channel, and Pt wire gently slide in the top and bottom groove (Reprinted from Reference [62] with permission).

Most microfluidic models use porous membrane segmentation to form sandwich structures in the chip that are similar to those used in transwell systems. Endothelial cells and other cells are cultured on two sides of a membrane that is placed at the interface of two microchannels to form a neural chamber and a vascular chamber [51]. Other researchers also used micro-gaps, trapezoidal structures or porous tubular structures to separate epithelial cells from other cells. Designs for microfluidic BBB models vary greatly. Figure 2 illustrates typical design of the models, and Table 1 shows the main characteristics for selected microfluidic models. These features will be discussed in detail below.

#### 3.1.1. Chip Material

Since microfluidic chips can be manufactured by different materials, making appropriate choices can not only reduce experimental costs, but also help improve the stability, sensitivity, and accuracy [46]. Silicon, the first-generation microfluidic device material, can be processed by MEMS technology to form microfluidic structures. However, the microfabrication process is usually expensive, time-consuming, and requires special microfabrication conditions [52].

Most microfluidic models now in use are polydimethylsiloxane (PDMS)-based systems because they have low cost and are easily microfabricated. PDMS is a polymeric organosilicon compound that is optically transparent, non-toxic, non-flammable, gas- and water-permeable [53]. Optical transparency is convenient for observation and photography during an experiment, while gas and water permeability are important for cell culturing in the microfluidic chip environment. Critically, PDMS chips can be prepared using a mask by the replica molding process. The replication step allows mass-production of chips from one mold. PDMS can tightly bind to glass, PDMS, and other materials after a simple plasma treatment [54]. It is therefore straightforward to design and fabricate various PDMS microfluidic chips to meet specific experimental requirements. Although PDMS devices are widely used in research, they have some limitations in the BBB studies. Native PDMS is hydrophobic and incompatible with most organic solvents, and hydrophobic molecules are easily adsorbed on the chip surface. Furthermore, the untreated PDMS surface has poor affinity for living cells. To reduce hydrophobicity and enhance cell adhesion during an experiment, it is important to modify the PDMS surface by plasma treatment or protein coating before initiating cell culture [55]. It has also been reported that uncrosslinked free PDMS monomers can leach out into the culture medium and affect cellular behavior [56,57].

To overcome the limitations of PDMS and make microfluidic chips more suitable for commercial applications, thermoplastics, such as polystyrene, cyclic olefin copolymer, and poly(methyl methacrylate) are used for microfluidic device fabrication. Thermoplastics have excellent light transmission performance and are more chemical stable, more compatible with organic solvents, and more acceptable to cell biologists. Using these materials, processed by lamination, embossing, and injection molding, microfluidic chip mass production is possible [81,82]. However, it is difficult to generate very complex microstructures in a thermoplastics-based microfluidic chip.

#### 3.1.2. Porous Membranes

In microfluidic models, porous membranes are used to separate the luminal and abluminal layers. The membranes provide the platform for co-culturing endothelial cells and other cells, and make permeability testing possible for models. The materials used for porous membranes include polycarbonate (PC), polyester (PE), polyethylene terephthalate, and polytetrafluoroethylene (PTFE). The selection of membrane materials should prioritize optical transparency and their capacity for cell adhesion under shear stress. Pore density, pore size, and membrane thickness may affect signal transduction between cells on different sides of the membrane. Most membranes used in experiments are about 10 μm thick and have 0.2 or 0.4 μm pores, yielding pore densities of 10^8^/mm^2^. PC membranes are the most widely used in microfluidic models, but are optically translucent, compromising the ability to visualize cells by light microscopy during an experiment. PE and PTFE membranes are transparent and can be used to monitor the formation of monolayer with phase contrast microscopy. However, since PE membranes have low adhesive strength, they tolerate shear stress for a limited period of time [83]. Sellgren found that PTFE membranes were more suitable than PE membranes for supporting cell attachment at realistic levels of shear stress [65]. Although endothelial cells and other cells can be co-cultured through porous membranes, the thickness of these artificial membranes restricts cell interaction and is relatively high compared with the thickness of the basement membrane in vivo [29].

#### 3.1.3. Endothelial Cells

Endothelial cells are the most important cells in the BBB. Initially, in vitro models used bovine, porcine, and rat primary BMECs, but the isolation of these cells is methodologically difficult and labor intensive. To decrease the time necessary to isolate primary cells, immortalized cell lines from diverse origins have been developed, but only a few of them exhibit the required barrier properties and functions. Lines that have been used in the BBB research include bEnd.3, a murine brain endothelial cell line [40,84], RBE4, a rat brain endothelial cell line [63,64], MDCK-MDR1, a line based on Mardin-Darby canine kidney cells transfected with the multidrug resistance gene MDR1 [85], and hCMEC/D3, a human adult cerebral microvascular endothelial cell line [72,76,78]. Compared with primary cells, immortalized cell lines can decrease the workload and reduce the time required to reach confluence. But some researchers report that immortalized cell lines cannot form complete tight junctions, resulting in a leaky barrier [86].

Most endothelial cells used in studies are from animal sources, which have species differences with human cells. Recently, human origin cells have been gradually used in model research. Adriani used human umbilical vein endothelial cells (HUVECs), co-cultured with rat astrocytes and neurons to form the BBB structure in microfluidic chip, and showed an intact monolayer and intercellular junctions [59]. In Yang’s research, HUVECs cell line ECV304 monoculture achieved higher TEER and lower permeability than bEnd3, but ECV304 monolayers lack the sufficient tightness and the evident of tight junction protein immunostaining, and when co-cultured with rat glioma C6 cells, barrier integrity is compromised [87]. Wang et al. derived BMECs from human induced pluripotent stem cells and co-cultured them with rat primary astrocytes. The TEER levels of this model peaked above 4000 Ω·cm^2^ and were sustained above 2000 Ω·cm^2^ for 10 days, which are the highest values reported in microfluidic models [74]. This model is closer to human physiology, not limited by cell availability, has the potential to be patient-specific, and holds great promise for drug permeability studies.

#### 3.1.4. Shear Stress

Flow in a microfluidic chip generates shear stress, as is the case for flow in natural environments. The shear stress in the microchannel has effect on cell growth, morphology, and cell function, and regulates gene expression and functional phenotypes in endothelial cells [88], and epithelial cells of different origins may exhibit different phenotypes under shear stress [73]. Exposure to fluid flow at physiological levels in a microfluidic chip increases the expression of tight junction proteins, enhances barrier integrity, and results in better barrier function. While low pathophysiological levels of fluid shear stress can stimulate the disintegration of tight junction [89]. In the physiological state, shear stress is 10–20 dynes·cm^−2^ in a 10 μm-diameter capillary [90]. In microfluidic chips, shear stress is mainly associated with fluid viscosity, flow rates through microchannels, channel geometry, and the flow profile. Due to the presence of cells and proteins, the viscosity of blood is greater than that of water. Flow rates in microfluidic models vary from 0.01 μL/min to 120 μL/min [65,91]. Therefore, it can be very important to analyze and predict shear stress in a microfluidic model using simulation software.

### 3.2. Assessment of Microfluidic Models

Using microfluidic models, it is possible to investigate cellular and molecular interactions. However, when implementing a new model, it is critical to assess the model’s performance. Most commonly, this involves testing for the presence of specific markers of tight junctions, measuring TEER, and observing the permeability of specific substances.

#### 3.2.1. Determining Specific Tight Junction Markers

In the BBB, cells have extensive tight junctions and adhesion molecules at interendothelial cell-cell junctions to maintain the barrier integrity [92,93]. Immunofluorescence or western blots can be used to observe the expression of specific markers, such as zona occludens-1 (ZO-1), claudin-5, and occludin. The P-glycoprotein efflux pump is another type of membrane transporter, and plays a vital function by preventing hydrophobic molecules from penetrating the BBB [94]. Expression of P-glycoprotein is also used to evaluate BBB characteristics in microfluidic models [64].

#### 3.2.2. Trans-Epithelial Electric Resistance (TEER) Measurement

TEER is a widely used parameter to monitor and evaluate barrier integrity and tightness. As epithelial cells are packed more tightly, fewer gaps exist in the barrier, which reduce the motion of ions and charged species and results in higher resistance. TEER is a quantitative measurement of the resistance over cell layers and cultured cell membranes [69]. If a microfluidic chip contains Ag/AgCl pellet electrodes, platinum electrodes, or some other device that can perform as an electrode, TEER measurement can be performed in real time [94]. The TEER value of an in vitro model should be as close as possible to the TEER in vivo, typically in the range of 1800 to 2000 Ω·cm^2^ [29], the in vitro values of most reported models are well below this range [95,96], 150 to 200 Ω·cm^2^ is considered the lowest acceptable TEER value in functional models [34].

TEER offers a fast, label-free, and real-time assessment of barrier tightness, but is not sufficient to judge barrier selectivity. TEER measurements on a chip are subject to several confounding factors. First, cell origin and the extent of cell confluence may affect resistance. Second, the distribution of current across the membrane interface in a chip may not be uniform, leading to overestimates of TEER. Third, TEER is sensitive to temperature and ionic composition of the culture medium. Therefore, these parameters need to be kept constant during the measurement process [97].

#### 3.2.3. Permeability Assessment

The BBB is a highly selective barrier that permits the passage of very few molecules. Small ions such as K^+^ and Cl^−^ can cross the BBB through ion channels. Small lipophilic molecules such as ethanol and nicotine can be transported passively across the barrier. Small polar molecules such as glucose, lactate, and pyruvate can cross the BBB by carrier-mediated transport. Finally, large molecules such as insulin, transferrin, leptin, albumin, and tumor necrosis factor alpha (TNFα) are passed through the BBB by receptor-mediated transport, adsorption-mediated transcytosis, and active efflux transporters [90,98].

To assess barrier function in a BBB model, permeability must be evaluated. A high quality model should have permeability characteristics similar to those found in vivo. When assessing permeability, it is important to select a suitable molecule. A good marker should be inert and have no effect on the physiology and function of the BBB. Fluorescein isothiocyanate (FITC) labeled dextrans, are widely used to evaluate the permeability [60,74,80]. Commercially available FITC-dextrans can be obtained with molecular weights of 4, 10, 40, and 70 kDa. ^14^C-D-mannitol (182 Da) and ^14^C-urea (60 Da) are also used in permeability experiments [35]. In most permeability analyses, only one marker is used. However, since different molecules traverse the barrier via different mechanisms, both hydrophilic and lipophilic molecules should be tested for permeability.

### 3.3. Application of Microfluidic Models

Microfluidic chip models exhibit unique advantages in the BBB research. First, the chips are easy to design and fabricate, and can be customized to meet specific experimental requirements. Microchannel sizes are similar to microvascular structures in vivo, and it is easy to achieve precise fluid control. Second, multi-dimensional network structures generate relatively independent and closed environments in the chip, and thus resemble the microenvironment in vivo. In microfluidic models, the cell behavior changes greatly from 2D to 3D, and can obtain more physiological information and prediction data [79]. Third, in microfluidic chips it is straightforward to combine and integrate various functional units. Consequently, the models can be used to observe cell morphology, image live cells for permeability studies, and monitor TEER in real time for barrier tightness using an integrated electrode.

Several microfluidic models have been developed in order to answer specific research questions. These models have been used to study the BBB function, screen drug candidates, and predict the clearance of pharmaceuticals by the BBB.

First, microfluidic models provide a new reliable platform to observe the influence of one or more molecules on the BBB characteristics. For example, Brown used the microfluidic model to study how the BBB responds to inflammatory stimuli, such as lipopolysaccharide or a cytokine cocktail. They found that inflammatory stimulation increases permeability, reduces the numbers of tight junctions, and alters the metabolomics profile [61]. Stimulation by a cytokine mix comprising TNF, interleukin-1b, interferon c, and LPS was reported to result in the loss of VE-cadherin and ZO-1 expression, indicating disruption of the endothelial barrier [66]. A model stimulated by 1 ng/mL TNF-α exhibited a 10-fold decrease in TEER [62]. Exposure to histamine caused an instantaneous transient drop in TEER, while permeability coefficients increased significantly at higher pH (>10) [37]. Perfusion of an astrocyte-conditioned medium improved the BBB function, increased the expression of tight junction molecules, and decreased permeability [64].

Microfluidic models can also be used to assess the permeability of different compounds, including free forms, binding to nanomaterials, or functionalized [79]. Falanga used a microfluidic model to evaluate a new nano drug delivery vector for the CNS, and found that nanoparticles pass more easily through the BBB barrier when combined with the membranotropic peptide GH625 [71]. Wevers evaluated the permeability of therapeutic antibodies by receptor-mediated endocytosis, antibody of human transferrin receptor (MEM-189) is easier to pass through the BBB compared with the control antibody [80]. Based on microfluidic models, Bonakdar also found sub-electroporation pulsed electric field can disrupt the integrity of the BBB and increase permeability [75]. In Papademetriou‘s research, he found that shear flow impacted the binding and internalization of angiopep-2 coupled liposomes nanoparticles by brain endothelial cells in microfluidic models [89].

## 4. Conclusions and Perspective

As the interface between the blood and the CNS, the BBB is critical for maintaining a steady state environment in the CNS. There is great interest in developing experimental models to understand the structural, physiological, and biochemical functions of the BBB, and to explore the mechanisms that change these properties under pathological conditions. Recently, microfluidic in vitro models have been established and evaluated. Microfluidic models are easy to design and fabricate, can simulate physiological fluid flow and shear stress, and can better mimic the BBB microenvironment compared with other models, and show great potential to further BBB research.

Although great progress has been made, microfluidic models are still in the early stages of development. It can be difficult to find a suitable model to meet all kinds of experimental requirements. Before these models are widely applied to the BBB-related research, several challenges must be overcome.

First, microfluidic models are not ready for commercial use. Microfluidic devices have the ability to control cell microenvironments and can be designed to meet specific requirements. However, simulation of the BBB microenvironment will require intricate systems, including complex microchannel networks with multiple elements such as pumps, valves, mixers, and detectors. As complexity increases, fabrication becomes more difficult. At the same time, minor changes and modifications to the models can produce chips with drastically different properties. Currently, most academic research use PDMS as the chip material, which is unfavorable for large-scale production for use in commercial applications [80]. Thermoplastics may be superior for industrial purposes, but it is more difficult to obtain complicated and meticulous microstructures using this material.

Second, even with advances in chip design and fabrication, there remains a large gap between the characteristics of microfluidic models and the in vivo environment. For example, in most experiments a porous membrane is used to separate epithelial cells and other cells, but the thickness of the membrane is 10 μm, which is over 300 times thicker than the naturally occurring basement membrane in endothelial cells. This large gap makes difficult for different types of cells to establish direct contact. To enable direct contact between co-cultured cells, microchannels can be filled with hydrogels as an extracellular matrix, but rigid extracellular matrix substrates have stiffness values orders of magnitude higher than those observed in living brain microvessels. In addition, while capillaries have an average diameter of 6–9 μm, modeled blood vessels are far larger due to the constraints imposed by processing technology. The BBB is also a complex and integrated system, while current microfluidic models are based mainly on only two or three kind of cells. In a co-culture system, it is difficult to distinguish paracrine and cell-cell contact mediated effects [99]. Moreover, the performance of a co-culture model is affected by many factors, such as cell type and origin, cell number and cell ratio, flow rates, and shear stress. All of these factors will need to be optimized.

Third, cell manipulation on a microfluidic chip requires substantial labor on the part of a researcher. There are six orders of magnitude differences between microfluidic chips and conventional laboratory equipment [100]. Many small operation details can affect model properties and experimental reproducibility, and unintended variations may result in widely different results. For example, it is difficult to seed cells at a particular place in a sealed chamber because bubbles in the channel can interfere with the process [101]. In order to improve experimental reproducibility, much more attention will be required to standardize details such as those involved in chip fabrication, cell seeding, cell localization, and other design factors. In order to compare the performance of different models, or compare data between in vitro models and in vivo experiments, it will also be necessary to standardize the measurement of parameters.

This review has presented recent progress in the design and evaluation of microfluidic in vitro BBB models, including advances in chip materials, porous membranes, the use of endothelial cells, the importance of shear stress, the detection specific markers to monitor tight junction formation and integrity, and measurements of TEER and permeability. However, like other models, microfluidic models cannot yet reproduce all the features of the BBB, and have limitations that must be addressed. By understanding and comparing the advantages and disadvantages of the design of different microfluidic models, we can choose the most suitable microfluidic models and experimental methods to adjust the data, so as to promote the BBB research. More distant goals are to optimize microfluidic models as screening platforms for candidate drugs and to apply them to study the pathogenesis in neurological diseases.

## Figures and Tables

**Table 1:** The main characters of some microfluidic blood-brain barrier (BBB) models.

Source	Main Character of Chip Design	Source of Endothelial Cells	Co-Cultured Cells	Character of Membranes	Protein Used for Channel Coating	Electrode in Chip	Markers Used in Tight Junction Determination	TEER Value of the Models	Molecule Used in Permeability Test	Application
Ref. [36]	16 independent function units, each unit consists of four uniform BBB regions, replicate the complex multicellular architecture, mechanical properties, 3D extracellular matrix	Primary rat BMECs	Primary rat astrocytes	No membrane	Rat tail type-I collagen	No electrode in chip, normal resistance meter	VE-Cadherin, ZO-1, Claunin-5, etc. (immunofluorescence staining)	1298 Ω∙cm^2^	Sodium fluorescein (376 Da)	Examination of brain metastasis and the therapeutic response of brain tumors
Ref. [40]	Three PDMS layers plus the PC membrane that divides the two chambers.	Primary human BMECs	Primary pericytes, Primary astrocytes and pluripotent stem cell -derived neurons	PC membrane (0.2 μm pores)	Laminin	23 ga stainless steel, not in chip	ZO-1 (immunofluorescence staining)	Reported in Ω/cm^2^, need to be converted	FITC-dextran (10 and 70 kDa)	Ascorbate transport across the BBB as an indication of active transport
Ref. [58]	Multi-layered channel structure made from patterned PDMS substrate with embedded electrode layers.	bEnd.3 cell line	Astrocytes C6 cell line	PC membrane (10 μm thick, 0.4 μm pores)	Poly-lysine and fibronectin	Two sets AgCl electrodes.	ZO-1 (immunofluorescence staining)	223–280 Ω∙cm^2^	Not used	Permeability of seven neuroactive drugs and TEER were quantified in models.
Ref. [59]	3 μm pores to allow diffusion of media and tracer between the central and outer compartments	HUVECs	Astrocytes CTX-TNA2 cell line	3 μm pores	Martrigel and fibronectin	No electrode	Not detected	Not measured	Texas red dextran (370 kDa) and rhodamine 123	Comparing permeability of three passive permeability markers and one marker subject to efflux
Ref. [60]	Two separate microchannels supply their respective co-culture tissues independently of one another, and can serve as the microenvironment of the outside and the inside of the BBB respectively.	HUVECs and human lung fibroblasts	Primary rat astrocyte and neurons	No membrane, using fibrin hydrogel	Fibrin hydrogel	No electrode	ZO-1. (immunofluorescence staining)	Not measured	FITC-dextran (20 kDa, 70 kDa)	A platform exhibits direct contact between neural and vascular tissues and a corresponding low permeability characteristic of in vivo BBB
Ref. [61]	Two-chamber system divided by PC membrane	Primary human BMECs	Human induced pluripotent stem cell-derived neurons and astrocytes	PC membrane (0.2 μm pores)	Laminin	Custom-built multifrequency impedance analyzer	ZO-1, claudin -5 (immunofluorescence staining)	Reported in Ω, need to be converted	FITC-dextran (10 kDa)	Understand responses to inflammatory stimulation
Ref. [62]	Small model, two-layer microchannel and membrane with platinum electrodes.	hCMEC/D3 cell line	No co-cultured cells	PC membrane (10 μm thick, 0.4 μm pores)	Collagen I	Platinum electrodes (diameter 200 μm)	ZO-1 (immunofluorescence staining)	36.9–120 Ω∙cm^2^	Not used	Observe shear stress and TNF-α on BBB function
Ref. [63]	Vascular conduit overlaid on top of a neural chamber separated by a PC membrane	RBE4 cell line	Mixture of neurons (4%), astrocytes (95%), and microglia (1%).	PC membrane (8 μm pores)	Poly-lysine and fibronectin	No electrode	ZO-1 (western blot)	Not measured	Alexafluor-dextran (3 kDa)	TNF-α simulation triggered neuroinflammation
Ref. [64]	The apical and basolateral side separated by 3 μm gaps formed by microfabricated pillars.	RBE4 cell line	No co-cultured cells	No membrane, micro-gaps (50 μm long, 3 μm wide, 3 μm deep) in PDMS wall	Fibronectin	No electrode	ZO-1, claudin (western blot) and P-glycoprotein protein efflux	Not measured	FITC- dextran (3–5 kDa)	Astrocyte-conditioned medium on BBB function
Ref. [65]	Two-compartment microfluidic devices were a membrane between two channels.	bEnd.3 cell line	C8D1A astrocytes cell line	PTFE or PE membrane (0.4 μm pores)	Fibronectin or collagen I	No electrode	Claudin-5 (immunofluorescence staining)	Not measured	FITC-dextran (70 kDa)	Study the optically transparent membrane used in models
Ref. [66]	Composed of an upper and a lower part that are combined with an adhesive film, three microchannel systems are integrated	hCMEC/D3 cell line	Mouse embryonic stem cells derived cortical spheroids	Polyethylene terephthalate membrane and PC membrane	Collagen A	No electrode	VE cadherin, ᵦ-catenin, ZO-1 (immunofluorescence staining)	Not measured	FITC-dextran (3 kDa)	Detect effects of neuroinflammation upon disruption of the endothelial layer in response to inflammatory signals.
Ref. [67]	The porous membrane is situated between the upper and the lower channels made of PDMS. This core is sandwiched by two glass slides with gold electrodes.	hCMEC/D3 cell line, primary rat brain endothelial cells	primary astrocytes and brain pericytes.	PET membrane, (23 μm thick, 0.45 μm pores, pore density 2 × 10^6^/cm^2^)	Rat tail collagen	A pair of 25-nm thick, transparent, gold electrodes was formed on each glass slide	ᵦ-catenin, ZO-1 (immunofluorescence staining)	monolayer: 28.5 ± 7.2 Ω∙cm^2^ Co-culture: 114.2 ± 35.7 Ω∙cm^2^	Sodium fluorescein (376 Da), FITC- dextran (4.4 kDa), Evans blue-labeled albumin (67 kDa)	Design a new device, which can co-culture of 3 types of cells, observe the cells by microscopy, monitor the TEER, and measure the monolayer permeability
Ref. [68]	Creating a cylindrical collagen gel containing a central hollow lumen inside a microchannel	Primary human BMECs	Primary human brain pericytes, primary human brain astrocytes	No membrane	Rat tail collagen I	No electrode	VEcadherin, ZO-1 (immunofluorescence staining)	Not measured	Alexa488- dextran (3 kDa)	Study the secretion profiles of G-CSF, IL-6 and IL-8 when the BBB stimulated with TNF-α
Ref. [69,70]	Two PDMS components are separated by PC membrane and form two-chamber system	hCMEC/D3 cell line	No co-cultured cells	PC membrane (0.4 μm pores)	Fibronectin	Four platinum wire electrodes inserted into two channels	ZO-1 (immunofluorescence staining)	22 ± 1.3 Ω∙cm^2^	Not used	Developed a stable and easily method to determine TEER in organ-on-chip applications.
Ref. [71]	Transparent polyester porous membrane sandwiched between a top and a bottom overlying channel made of PMMA.	bEnd.3 cell line	No co-cultured cells	Polyester membrane (3 μm pores)	Not used	Platinum electrodes	Claudin-5 (immunofluorescence staining)	About 1000 Ω∙cm^2^	FITC- bovine serum albumin	Test the ability of a peptide to transport nanoparticles across BBB under flow conditions.
Ref. [72]	Two central hydrogel regions for co-culturing astrocytes and neurons, two side channels for hosting endothelial cells and media.	HUVECs and hCMEC/D3 cell line	Primary rat neurons and astrocytes	No membrane, separated by 9 trapezoidal structures	Poly-lysine and collagen I	No electrode	ZO-1 (immunofluorescence staining)	Not measured	Oregon green 488- dextran (10 kDa), Texas red dextran (70 kDa)	Compounds and factors on neural growth and maturation
Ref. [73]	Four rectangular channels with different heights to allow simultaneous measurements at different shear stresses.	Human BMECs from the BC1 human induced pluripotent stem cell line	No co-cultured cells	No membrane	Fibronectin and collagen IV	No electrode	claudin-5, occludin, and ZO-1 (immunofluorescence staining)	Not measured	Not used	Study the role of shear stress in modulating the character of human brain microvascular endothelial cells derived from induced pluripotent stem cells.
Ref. [74]	Consists of a cell insert and three 3D printed plastic layers with two electrodes	BMECs from human induced pluripotent stem cells	Primary rat astrocytes	PC membrane (0.4 μm pores)	Collagen IV and fibronectin	Two 0.8 mm diameter Ag/AgCl pellet electrode	ZO-1, Claunin-5. (immunofluorescence staining)	Peaked above 4000 Ω·cm^2^, sustained above 2000 Ω·cm^2^	FITC-dextran (70, 20 and 4 kDa), Caffeine, cimetidine, and doxorubicin	Model research
Ref. [75]	A double layer microfluidic device with an embedded membrane, the top layer contains a single channel, the bottom channel contains an array of 6 channels	hCMEC	No co-cultured cells	Polyester membrane (0.4 μm pores)	Fibronectin	No electrode	Not detected	Not measured	fluorescent sodium salt (376 Da), FITC- dextran (70 kDa)	Pulsed electric fields may enhance drug delivery to the brain by disrupting the integrity of the BBB and allowing otherwise impermeable drugs to reach target areas.
Ref. [76]	Two isolated compartments with the hydrogel reservoir	hCMEC/D3 cell line	p5–p7 normal human astrocytes	No membrane	Collagen I, matrigel, hyaluronan	No electrode in chip, normal impedance spectroscopy	ZO-1. (immunofluorescence staining)	Static condition: about 200 Ω·cm^2^ flow condition: about 1000 Ω·cm^2^	FITC-dextran (4 kDa)	Indicated that the mechanical stress exerted by blood flow is an important regulator of transport both across and along the walls of cerebral microvasculature.
Ref. [77]	A 4 × 4 intersecting microchannel array forms 16 BBB sites on a chip, with a multielectrode array integrated to measure the TEER from all 16 different sites.	Primary mouse BMECs	Primary mouse astrocytes	PC membrane (10 μm thick, 0.4 μm pores)	Fibronectin or matrigel	Multielectrode arrays, a thin titanium adhesion layer and a gold layer	ZO-1. (immunofluorescence staining)	Reported in Ω, need to be converted	Texas Red dextran (3 kDa), Alexa 546 dextran (10 kDa), FITC dextran (70 kDa)	Developed multisite BBB chip is expected to be used for screening drug by more accurately predicting their permeability through BBB as well as their toxicity.
Ref. [78]	Microchannel with temporary chitosan-based membrane	hCMEC/D3 cell line	P6-P10 human astrocytes from the cerebral cortex	temporary chitosan-based membrane	Hydrogel matrigel	No electrode	Not detected	Not measured	Not used	To obtain a co-culture without a nonphysiological membrane making use of a temporary chitosan membrane in a microfluidic channel.
Ref. [79]	Bio-printing, 10 μm average diameter tubes encasing a liquid flow having around 1 mm·s^−^^1^ average speed. On the surface of each tube regular pores allowing for mass transport.	bEnd.3 cell line	U87 glioblastoma cells	No membrane, porous tubular structures on tube surface (pore diameter: 1 μm)	Not used	No electrode in chip, commercially Voltohmmeter with two electrodes	ZO-1. (immunofluorescence staining)	75 ± 2 Ω∙cm^2^	Dextran	Presented a dynamic 3D biohybrid model of the BBB able to reproduce at 1:1 scale the capillaries of the neurovascular system.
Ref. [80]	High-throught, the model harbors 96 or 40 chips in a 384-well plate. In each chip, a perfused vessel of BMECs was grown against an extracellular matrix gel, astrocytes and pericytes were added on the other side of the gel to complete the BBB model.	Human TY10 cell line (isolated from normal brain tissue from a patient with meningioma)	Human hBPCT cell line pericytes from brain tissue of a patient t. Human hAst cell line astrocytes from human primary astrocytes distributed by Lonza.	No artificial membranes, using extracellular matrix gel	Collagen-I	No electrode	claudin-5, VE-cadherin, PECAM-1 (immunofluorescence staining)	Not measured	FITC-dextran (20 kDa)	Developed a high-throughput plate-based model, and used to assess passage of large biopharmaceuticals across the BBB.

## References

[1] Cai Z., Qiao P.F., Wan C.Q., Cai M., Zhou N.K., Li Q. (2018). Role of Blood-Brain Barrier in Alzheimer’s Disease. J. Alzheimers Dis..

[2] Vargas-Osorio Z., Da Silva-Candal A., Piñeiro Y., Iglesias-Rey R., Sobrino T., Campos F., Castillo J., Rivas J. (2019). Multifunctional Superparamagnetic Stiff Nanoreservoirs for Blood Brain Barrier Applications. Nanomaterials.

[3] Shimizu F., Nishihara H., Kanda T. (2018). Blood-brain barrier dysfunction in immuno-mediated neurological diseases. Immunol. Med..

[4] Sharma G., Sharma A.R., Lee S.S., Bhattacharya M., Nam J.S., Chakraborty C. (2019). Advances in nanocarriers enabled brain targeted drug delivery across blood brain barrier. Int. J. Pharm..

[5] Abdullahi W., Tripathi D., Ronaldson P.T. (2018). Blood-brain barrier dysfunction in ischemic stroke: Targeting tight junctions and transporters for vascular protection. Am. J. Physiol. Cell Physiol..

[6] Li J., Li C., Yuan W., Wu J., Li J., Li Z., Zhao Y. (2017). Mild hypothermia alleviates brain oedema and blood-brain barrier disruption by attenuating tight junction and adherens junction breakdown in a swine model of cardiopulmonary resuscitation. PLoS ONE.

[7] Bhowmick S., D’Mello V., Caruso D., Wallerstein A., Abdul-Muneer P.M. (2019). Impairment of pericyte-endothelium crosstalk leads to blood-brain barrier dysfunction following traumatic brain injury. Exp. Neurol..

[8] Rajagopal N., Irudayanathan F.J., Nangia S. (2019). Palmitoylation of Claudin-5 Proteins Influences Their Lipid Domain Affinity and Tight Junction Assembly at the Blood-Brain Barrier Interface. J. Phys. Chem. B.

[9] Iadecola C. (2017). The Neurovascular Unit Coming of Age: A Journey through Neurovascular Coupling in Health and Disease. Neuron.

[10] Ozaki T., Nakamura H., Kishima H. (2019). Therapeutic strategy against ischemic stroke with the concept of neurovascular unit. Neurochem. Int..

[11] Guerra M., Blázquez J.L., Rodríguez E.M. (2017). Blood-brain barrier and foetal-onset hydrocephalus, with a view on potential novel treatments beyond managing CSF flow. Fluids Barriers CNS.

[12] Singh A., Kim W., Kim Y., Jeong K., Kang C.S., Kim Y., Koh J., Mahajan S.D., Prasad P.N., Kim S. (2016). Multifunctional Photonics Nanoparticles for Crossing the Blood-Brain Barrier and Effecting Optically Trackable Brain Theranostics. Adv. Funct. Mater..

[13] Miranda A., Cova T., Sousa J., Vitorino C., Pais A. (2018). Computational modeling in glioblastoma: From the prediction of blood-brain barrier permeability to the simulation of tumor behavior. Future Med. Chem..

[14] Rüber T., David B., Lüchters G., Nass R.D., Friedman A., Surges R., Stöcker T., Weber B., Deichmann R., Schlaug G. (2018). Evidence for peri-ictal blood-brain barrier dysfunction in patients with epilepsy. Brain.

[15] Merali Z., Huang K., Mikulis D., Silver F., Kassner A. (2017). Evolution of blood-brain-barrier permeability after acute ischemic stroke. PLoS ONE.

[16] Sweeney M.D., Sagare A.P., Zlokovic B.V. (2018). Blood-brain barrier breakdown in Alzheimer disease and other neurodegenerative disorders. Nat. Rev. Neurol..

[17] Akaishi T., Takahashi T., Nakashima I. (2018). Oligoclonal bands and periventricular lesions in multiple sclerosis will not increase blood-brain barrier permeability. J. Neurol. Sci..

[18] Galla H.J. (2018). Monocultures of primary porcine brain capillary endothelial cells: Still a functional in vitro model for the blood-brain-barrier. J. Control. Release.

[19] Alluri H., Shaji C.A., Davis M.L., Tharakan B.A. (2018). Mouse Controlled Cortical Impact Model of Traumatic Brain Injury for Studying Blood-Brain Barrier Dysfunctions. Methods Mol. Biol..

[20] Zhong Z., Li Z., Chakrabarty K., Ho T.Y., Lee C.Y. (2019). Micro-Electrode-Dot-Array Digital Microfluidic Biochips: Technology, Design Automation, and Test Techniques. IEEE Trans. Biomed. Circuits Syst..

[21] Alam M.K., Koomson E., Zou H., Yi C., Li C.W., Xu T., Yang M. (2018). Recent advances in microfluidic technology for manipulation and analysis of biological cells (2007–2017). Anal. Chim. Acta.

[22] Chin E., Goh E. (2018). Blood-brain barrier on a chip. Methods Cell Biol..

[23] Kuhnline Sloan C.D., Nandi P., Linz T.H., Aldrich J.V., Audus K.L., Lunte S.M. (2012). Analytical and biological methods for probing the blood-brain barrier. Annu. Rev. Anal. Chem.

[24] Betzer O., Shilo M., Opochinsky R., Barnoy E., Motiei M., Okun E., Yadid G., Popovtzer R. (2017). The effect of nanoparticle size on the ability to cross the blood-brain barrier: An in vivo study. Nanomedicine.

[25] Perrin S. (2017). Preclinical research: Make mouse studies work. Nature.

[26] Pandey P.K., Sharma A.K., Gupta U. (2015). Blood brain barrier: An overview on strategies in drug delivery, realistic in vitro modeling and in vivo live tracking. Tissue Barriers.

[27] Gaston J.D., Bischel L.L., Fitzgerald L.A., Cusick K.D., Ringeisen B.R., Pirlo R.K. (2017). Gene Expression Changes in Long-Term In Vitro Human Blood-Brain Barrier Models and Their Dependence on a Transwell Scaffold Material. J. Healthc. Eng..

[28] Hatherell K., Couraud P.O., Romero I.A., Weksler B., Pilkington G.J. (2011). Development of a three-dimensional, all-human in vitro model of the blood-brain barrier using mono-, co-, and tri-cultivation Transwell models. J. Neurosci. Methods.

[29] Kaisar M.A., Sajja R.K., Prasad S., Abhyankar V.V., Liles T., Cucullo L. (2017). New experimental models of the blood-brain barrier for CNS drug discovery. Expert Opin. Drug Discov..

[30] Czupalla C.J., Liebner S., Devraj K. (2014). In vitro models of the blood-brain barrier. Methods Mol. Biol..

[31] Humpel C. (2015). Organotypic brain slice cultures: A review. Neuroscience.

[32] Morin-Brureau M., De Bock F., Lerner-Natoli M. (2013). Organotypic brain slices: A model to study the neurovascular unit micro-environment in epilepsies. Fluids Barriers CNS.

[33] Alcendor D.J., Block F.E., Cliffel D.E., Daniels J.S., Ellacott K.L., Goodwin C.R., Hofmeister L.H., Li D., Markov D.A., May J.C. (2013). Neurovascular unit on a chip: Implications for translational applications. Stem Cell Res. Ther..

[34] Wolff A., Antfolk M., Brodin B., Tenje M. (2015). In Vitro Blood-Brain Barrier Models-An Overview of Established Models and New Microfluidic Approaches. J. Pharm. Sci..

[35] Wang J.D., Khafagy E.S., Khanafer K., Takayama S., ElSayed M.E. (2016). Organization of Endothelial Cells, Pericytes, and Astrocytes into a 3D Microfluidic in Vitro Model of the Blood-Brain Barrier. Mol. Pharm..

[36] Xu H., Li Z., Yu Y., Sizdahkhani S., Ho W.S., Yin F., Wang L., Zhu G., Zhang M., Jiang L. (2016). A dynamic in vivo-like organotypic blood-brain barrier model to probe metastatic brain tumors. Sci. Rep..

[37] Booth R., Kim H. (2012). Characterization of a microfluidic in vitro model of the blood-brain barrier (μBBB). Lab Chip.

[38] Gao Z., Chen Y., Cai X., Xu R. (2017). Predict drug permeability to blood-brain-barrier from clinical phenotypes: Drug side effects and drug indications. Bioinformatics.

[39] Toropov A.A., Toropova A.P., Beeg M., Gobbi M., Salmona M. (2017). QSAR model for blood-brain barrier permeation. J. Pharmacol. Toxicol. Methods.

[40] Brown J.A., Pensabene V., Markov D.A., Allwardt V., Neely M.D., Shi M., Britt C.M., Hoilett O.S., Yang Q., Brewer B.M. (2015). Recreating blood-brain barrier physiology and structure on chip: A novel neurovascular microfluidic bioreactor. Biomicrofluidics.

[41] Virumbrales-Muñoz M., Ayuso J.M., Lacueva A., Randelovic T., Livingston M.K., Beebe D.J., Oliván S., Pereboom D., Doblare M., Fernández L. (2019). Enabling cell recovery from 3D cell culture microfluidic devices for tumour microenvironment biomarker profiling. Sci. Rep..

[42] Shang M., Soon R.H., Lim C.T., Khoo B.L., Han J. (2019). Microfluidic modelling of the tumor microenvironment for anti-cancer drug development. Lab Chip.

[43] Michna R., Gadde M., Ozkan A., DeWitt M., Rylander M. (2018). Vascularized microfluidic platforms to mimic the tumor microenvironment. Biotechnol. Bioeng..

[44] Smith G.D., Takayama S. (2017). Application of microfluidic technologies to human assisted reproduction. Mol. Hum. Reprod..

[45] Kou S., Cheng D., Sun F., Hsing I.M. (2016). Microfluidics and microbial engineering. Lab Chip.

[46] Liu Z., Han X., Qin L. (2016). Recent Progress of Microfluidics in Translational Applications. Adv. Healthc. Mater..

[47] An F., Qu Y., Liu X., Zhong R., Luo Y. (2015). Organ-on-a-Chip: New Platform for Biological Analysis. Anal. Chem. Insights.

[48] Sivandzade F., Cucullo L. (2018). In-vitro blood-brain barrier modeling: A review of modern and fast-advancing technologies. J. Cereb. Blood Flow Metab..

[49] Thompson A.J., Ma L.J., Plegue T.J., Potkay J.A. (2019). Design Analysis and Optimization of a Single-Layer PDMS Microfluidic Artificial Lung. IEEE Trans. Biomed. Eng..

[50] Wilmer M.J., Ng C.P., Lanz H.L., Vulto P., Suter-Dick L., Masereeuw R. (2016). Kidney-on-a-Chip Technology for Drug-Induced Nephrotoxicity Screening. Trends. Biotechnol..

[51] Wilhelm I., Krizbai I.A. (2014). In vitro models of the blood-brain barrier for the study of drug delivery to the brain. Mol. Pharm..

[52] Faustino V., Catarino S.O., Lima R., Minas G. (2016). Biomedical microfluidic devices by using low-cost fabrication techniques: A review. J. Biomech..

[53] Takano A., Ogawa T., Tanaka M., Futai N. (2011). On-chip incubation system for long-term microfluidic cell culture. Conf. Proc. IEEE Eng. Med. Biol. Soc..

[54] Jenkins G. (2013). Rapid prototyping of PDMS devices using SU-8 lithography. Methods Mol. Biol..

[55] Menon N.V., Chuah Y.J., Cao B., Lim M., Kang Y. (2014). A microfluidic co-culture system to monitor tumor-stromal interactions on a chip. Biomicrofluidics.

[56] Du G., Fang Q., den Toonder J.M. (2016). Microfluidics for cell-based high throughput screening platforms—A review. Anal. Chim. Acta.

[57] Wong I., Ho C.M. (2009). Surface molecular property modifications for poly(dimethylsiloxane) (PDMS) based microfluidic devices. Microfluid. Nanofluid..

[58] Booth R., Kim H. (2014). Permeability analysis of neuroactive drugs through a dynamic microfluidic in vitro blood-brain barrier model. Ann. Biomed. Eng..

[59] Terrell-Hall T.B., Ammer A.G., Griffith J.I., Lockman P.R. (2017). Permeability across a novel microfluidic blood-tumor barrier model. Fluids Barriers CNS.

[60] Bang S., Lee S.R., Ko J., Son K., Tahk D., Ahn J., Im C., Jeon N.L. (2017). A Low Permeability Microfluidic Blood-Brain Barrier Platform with Direct Contact between Perfusable Vascular Network and Astrocytes. Sci. Rep..

[61] Brown J.A., Codreanu S.G., Shi M., Sherrod S.D., Markov D.A., Neely M.D., Britt C.M., Hoilett O.S., Reiserer R.S., Samson P.C. (2016). Metabolic consequences of inflammatory disruption of the blood-brain barrier in an organ-on-chip model of the human neurovascular unit. J. Neuroinflamm..

[62] Griep L.M., Wolbers F., de Wagenaar B., ter Braak P.M., Weksler B.B., Romero I.A., Couraud P.O., Vermes I., van der Meer A.D., van den Berg A. (2013). BBB on chip: Microfluidic platform to mechanically and biochemically modulate blood-brain barrier function. Biomed. Microdevices.

[63] Achyuta A.K., Conway A.J., Crouse R.B., Bannister E.C., Lee R.N., Katnik C.P., Behensky A.A., Cuevas J., Sundaram S.S. (2013). A modular approach to create a neurovascular unit-on-a-chip. Lab Chip.

[64] Prabhakarpandian B., Shen M.C., Nichols J.B., Mills I.R., Sidoryk-Wegrzynowicz M., Aschner M., Pant K. (2013). SyM-BBB: A microfluidic Blood Brain Barrier model. Lab Chip.

[65] Sellgren K.L., Hawkins B.T., Grego S. (2015). An optically transparent membrane supports shear stress studies in a three-dimensional microfluidic neurovascular unit model. Biomicrofluidics.

[66] Raasch M., Rennert K., Jahn T., Gärtner C., Schönfelder G., Huber O., Seiler A.E., Mosig A.S. (2016). An integrative microfluidically supported in vitro model of an endothelial barrier combined with cortical spheroids simulates effects of neuroinflammation in neocortex development. Biomicrofluidics.

[67] Walter F.R., Valkai S., Kincses A., Petneházi A., Czeller T., Veszelka S., Ormos P., Deli M.A., Dér A. (2016). A versatile lab-on-a-chip tool for modeling biological barriers. Sens. Actuators B.

[68] Herland A., van der Meer A.D., FitzGerald E.A., Park T.E., Sleeboom J.J., Ingber D.E. (2016). Distinct Contributions of Astrocytes and Pericytes to Neuroinflammation Identified in a 3D Human Blood-Brain Barrier on a Chip. PLoS ONE.

[69] Van der Helm M.W., Odijk M., Frimat J.P., van der Meer A.D., Eijkel J.C.T., van den Berg A., Segerink L.I. (2016). Direct quantification of transendothelial electrical resistance in organs-on-chips. Biosens. Bioelectron..

[70] van der Helm M.W., Odijk M., Frimat J.P., van der Meer A.D., Eijkel J.C.T., van den Berg A., Segerink L.I. (2017). Fabrication and Validation of an Organ-on-chip System with Integrated Electrodes to Directly Quantify Transendothelial Electrical Resistance. J. Vis. Exp..

[71] Falanga A.P., Pitingolo G., Celentano M., Cosentino A., Melone P., Vecchione R., Guarnieri D., Netti P.A. (2017). Shuttle-mediated nanoparticle transport across an in vitro brain endothelium under flow conditions. Biotechnol. Bioeng..

[72] Adriani G., Ma D., Pavesi A., Kamm R.D., Goh E.L. (2017). A 3D neurovascular microfluidic model consisting of neurons, astrocytes and cerebral endothelial cells as a blood-brain barrier. Lab Chip.

[73] DeStefano J.G., Xu Z.S., Williams A.J., Yimam N., Searson P.C. (2017). Effect of shear stress on iPSC-derived human brain microvascular endothelial cells (dhBMECs). Fluids Barriers CNS.

[74] Wang Y.I., Abaci H.E., Shuler M.L. (2017). Microfluidic blood-brain barrier model provides in vivo-like barrier properties for drug permeability screening. Biotechnol. Bioeng..

[75] Bonakdar M., Graybill P.M., Davalos R.V. (2017). A microfluidic model of the blood-brain barrier to study permeabilization by pulsed electric fields. RSC Adv..

[76] Partyka P.P., Godsey G.A., Galie J.R., Kosciuk M.C., Acharya N.K., Nagele R.G., Galie P.A. (2017). Mechanical stress regulates transport in a compliant 3D model of the blood-brain barrier. Biomaterials.

[77] Jeong S., Kim S., Buonocore J., Park J., Welsh C.J., Li J., Han A. (2018). A Three-Dimensional Arrayed Microfluidic Blood-Brain Barrier Model with Integrated Electrical Sensor Array. IEEE Trans. Biomed. Eng..

[78] Tibbe M.P., Leferink A.M., van den Berg A., Eijkel J.C.T., Segerink L.I. (2018). Microfluidic Gel Patterning Method by Use of a Temporary Membrane for Organ-On-Chip Applications. Adv. Mater. Technol..

[79] Marino A., Tricinci O., Battaglini M., Filippeschi C., Mattoli V., Sinibaldi E., Ciofani G. (2018). A 3D Real-Scale, Biomimetic, and Biohybrid Model of the Blood-Brain Barrier Fabricated through Two-Photon Lithography. Small.

[80] Wevers N.R., Kasi D.G., Gray T., Wilschut K.J., Smith B., van Vught R., Shimizu F., Sano Y., Kanda T., Marsh G. (2018). A perfused human blood-brain barrier on-a-chip for high-throughput assessment of barrier function and antibody transport. Fluids Barriers CNS.

[81] Sahore V., Doonan S.R., Bailey R.C. (2018). Droplet Microfluidics in Thermoplastics: Device Fabrication, Droplet Generation, and Content Manipulation using Integrated Electric and Magnetic Fields. Anal. Methods.

[82] Matellan C., Del Río Hernández A.E. (2018). Cost-effective rapid prototyping and assembly of poly(methyl methacrylate) microfluidic devices. Sci. Rep..

[83] Shao J., Wu L., Wu J., Zheng Y., Zhao H., Jin Q., Zhao J. (2009). Integrated microfluidic chip for endothelial cells culture and analysis exposed to a pulsatile and oscillatory shear stress. Lab Chip.

[84] Fotticchia I., Guarnieri D., Fotticchia T., Falanga A.P., Vecchione R., Giancola C., Netti P.A. (2016). Energetics of ligand-receptor binding affinity on endothelial cells: An in vitro model. Colloids Surf. B Biointerfaces.

[85] Yang B., Du S., Lu Y., Jia S., Zhao M., Bai J., Li P., Wu H. (2018). Influence of paeoniflorin and menthol on puerarin transport across MDCK and MDCK-MDR1 cells as blood-brain barrier in vitro model. J. Pharm. Pharmacol..

[86] Henderson J.T., Piquette-Miller M. (2015). Blood-brain barrier: An impediment to neuropharmaceuticals. Clin. Pharmacol. Ther..

[87] Yang S., Mei S., Jin H., Zhu B., Tian Y., Huo J., Cui X., Guo A., Zhao Z. (2017). Identification of two immortalized cell lines, ECV304 and bEnd3, for in vitro permeability studies of blood-brain barrier. PLoS ONE.

[88] Cucullo L., Hossain M., Puvenna V., Marchi N., Janigro D. (2011). The role of shear stress in Blood-Brain Barrier endothelial physiology. BMC Neurosci..

[89] Papademetriou I., Vedula E., Charest J., Porter T. (2018). Effect of flow on targeting and penetration of angiopep- decorated nanoparticles in a microfluidic model blood-brain barrier. PLoS ONE.

[90] Wong A.D., Ye M., Levy A.F., Rothstein J.D., Bergles D.E., Searson P.C. (2013). The blood-brain barrier: An engineering perspective. Front. Neuroeng..

[91] Deosarkar S.P., Prabhakarpandian B., Wang B., Sheffield J.B., Krynska B., Kiani M.F. (2015). A Novel Dynamic Neonatal Blood-Brain Barrier on a Chip. PLoS ONE.

[92] Tang Z., Guo D., Xiong L., Wu B., Xu X., Fu J., Kong L., Liu Z., Xie C. (2018). TLR4/PKCα/occludin signaling pathway may be related to blood-brain barrier damage. Mol. Med. Rep..

[93] Greene C., Hanley N., Campbell M. (2019). Claudin-5: Gatekeeper of neurological function. Fluids Barriers CNS.

[94] De Lange E.C.M., Vd Berg D.J., Bellanti F., Voskuyl R.A., Syvänen S. (2018). P-glycoprotein protein expression versus functionality at the blood-brain barrier using immunohistochemistry, microdialysis and mathematical modeling. Eur. J. Pharm. Sci..

[95] Srinivasan B., Kolli A.R., Esch M.B., Abaci H.E., Shuler M.L., Hickman J.J. (2015). TEER measurement techniques for in vitro barrier model systems. J. Lab. Autom..

[96] Hajal C., Campisi M., Mattu C., Chiono V., Kamm R.D. (2018). In vitro models of molecular and nano-particle transport across the blood-brain barrier. Biomicrofluidics.

[97] Odijk M., van der Meer A.D., Levner D., Kim H.J., van der Helm M.W., Segerink L.I., Frimat J.P., Hamilton G.A., Ingber D.E., van den Berg A. (2015). Measuring direct current trans-epithelial electrical resistance in organ-on-a-chip microsystems. Lab Chip.

[98] Patel M.M., Patel B.M. (2017). Crossing the Blood-Brain Barrier: Recent Advances in Drug Delivery to the Brain. CNS Drugs.

[99] Battiston K.G., Cheung J.W., Jain D., Santerre J.P. (2014). Biomaterials in co-culture systems: Towards optimizing tissue integration and cell signaling within scaffolds. Biomaterials.

[100] Geraili A., Jafari P., Hassani M.S., Araghi B.H., Mohammadi M.H., Ghafari A.M., Tamrin S.H., Modarres H.P., Kolahchi A.R., Ahadian S. (2017). Controlling Differentiation of Stem Cells for Developing Personalized Organ-on-Chip Platforms. Adv. Healthc. Mater..

[101] Tazawa H., Sunaoshi S., Tokeshi M., Kitamori T., Ohtani-Kaneko R. (2016). An Easy-to-Use Polystyrene Microchip-based Cell Culture System. Anal. Sci..

